# Supplementary material for: GROMTools: scalable individual-level GReX imputation for mega-biobank-scale cohorts
Source: medRxiv. 2026 Apr 28:2026.04.27.26351635. Preprint. [Version 1] doi: 10.64898/2026.04.27.26351635 (PMC13142567; doi:10.64898/2026.04.27.26351635)
Supplement: Supplement 1 [file media-1.pdf]

Supplementary Material

GROMTools: scalable individual-level GReX imputation for mega-biobank-scale cohorts

Authors: Marios Anyfantakis[1-7], Sanan Venkatesh[1-6], Jamie Bennett[1-6], Gabriel E. Hoffman[1-6], Panos Roussos[1-6], Georgios Voloudakis[1-6,8]

Affiliations:
[1]Department of Psychiatry, Icahn School of Medicine at Mount Sinai, New York, NY, USA
[2]Center for Disease Neurogenomics, Icahn School of Medicine at Mount Sinai, New York, NY USA
[3]Friedman Brain Institute, Icahn School of Medicine at Mount Sinai, New York, NY, USA
[4]Department of Genetics and Genomic Science, Icahn School of Medicine at Mount Sinai, New York, NY, USA
[5]Mental Illness Research, Education, and Clinical Center (VISN 2 South), James J. Peters VA Medical Center, Bronx, NY, USA
[6]Center for Precision Medicine and Translational Therapeutics, James J. Peters VA Medical Center, Bronx, NY, USA.
[7]University of Crete School of Medicine, University Hospital of Heraklion, Heraklion, Crete, Greece
[8]Department of Artificial Intelligence and Human Health, Icahn School of Medicine at Mount Sinai, New York, NY, USA

SUPPLEMENTARY TABLES 3
Supplementary Table 1 3
Supplementary Table 2 3
Supplementary Table 3 4

SUPPLEMENTARY TABLES

Supplementary Table 1

Method	Sample size	CPU time (sec)	Peak memory (MB)
**GROMTools**	50,000	91.53	1,017
**GROMTools**	100,000	168.90	1,209
**GROMTools**	150,000	295.58	1,472
**GROMTools**	250,000	577.11	1,753
**GROMTools**	350,000	604.49	2,163
**GROMTools**	450,000	748.37	2,543
**PLINK2**	50,000	17,648.17	15,720
**PLINK2**	100,000	35,257.94	29,267
**PLINK2**	150,000	54,028.79	42,829
**PLINK2**	250,000	62,862.50	69,936
**PLINK2**	350,000	88,437.69	97,046
**PLINK2**	450,000	113,614.30	124,144
**PrediXcan**	50,000	10,275.93	15,383
**PrediXcan**	100,000	20,186.21	28,912
**PrediXcan**	150,000	31,367.30	42,458
**PrediXcan**	250,000	39,831.50	69,542
**PrediXcan**	350,000	70,195.68	96,611
**PrediXcan**	450,000	90,841.42	123,656

Supplementary Table 1. Comparison of CPU time and peak memory usage across benchmark sample sizes for GROMTools, PLINK2, and PrediXcan.

Supplementary Table 2

Method	Sample Size	Read-like I/O bytes (rchar)	Write-like I/O bytes (wchar)
**GROMTools**	50,000	8.80E+08	4.70E+09
**GROMTools**	100,000	1.75E+09	9.39E+09
**GROMTools**	150,000	2.62E+09	1.41E+10
**PLINK2**	50,000	3.16E+10	2.99E+10
**PLINK2**	100,000	5.54E+10	5.63E+10
**PLINK2**	150,000	7.92E+10	8.28E+10
**PrediXcan**	50,000	1.27E+10	1.37E+10
**PrediXcan**	100,000	2.43E+10	2.75E+10
**PrediXcan**	150,000	3.59E+10	4.12E+10

Supplementary Table 2. Read-like and write-like I/O volume across benchmark sample sizes for GROMTools, PLINK2, and PrediXcan.

Supplementary Table 3

Model
Excitatory Neuron
Inhibitory Neuron
Immune Cell
Mural Cell
Endothelial Cell
Astrocyte
Oligodendrocyte
Oligodendrocyte Progenitor Cell
Layer 2-3 Intratelencephalic Excitatory Neuron
Layer 3-5 Intratelencephalic Excitatory Neuron 1
Layer 3-5 Intratelencephalic Excitatory Neuron 2
Layer 3-5 Intratelencephalic Excitatory Neuron 3
Layer 5-6 Near-Projecting Excitatory Neuron
Layer 5 Extratelencephalic Excitatory Neuron
Layer 6 Corticothalamic Excitatory Neuron
Layer 6 Intratelencephalic Excitatory Neuron 1
Layer 6 Intratelencephalic Excitatory Neuron 2
Layer 6B Excitatory Neuron
ADARB2 Inhibitory Neuron
Ivy cell (LAMP5 LHX6 Inhibitory Neuron)
Neurogliaform cell (LAMP5 RELN Inhibitory Neuron)
Basket Cell (PVALB Inhibitory Neuron)
Chandelier Cell (PVALB CHC Inhibitory Neuron)
Martinotti and Non-Martinotti cell (SST Inhibitory Neuron)
VIP Inhibitory Neuron
Microglia
Perivascular Macrophage
Adaptive Immune Cell
Pericyte
Smooth Muscle Cell
Vascular Leptomeningeal Cell
Excitatory Neuron
Inhibitory Neuron

Supplementary Table 3. Transcriptomic imputation models included in the benchmarking analyses. Only European ancestry models were utilized. Source: Venkatesh et al., 2026.
